# Effect of oral consumption of capsules containing *Lactobacillus paracasei* LPC-S01 on the vaginal microbiota of healthy adult women: a randomized, placebo-controlled, double-blind crossover study

**DOI:** 10.1093/femsec/fiaa084

**Published:** 2020-05-08

**Authors:** Ranjan Koirala, Giorgio Gargari, Stefania Arioli, Valentina Taverniti, Walter Fiore, Elena Grossi, Gaia Maria Anelli, Irene Cetin, Simone Guglielmetti

**Affiliations:** 1 Division of Food Microbiology and Bioprocesses, Department of Food, Environmental and Nutritional Sciences, University of Milan, via Luigi Mangiagalli 25, 20133, Milan, Italy; 2 Sofar S.p.A., Via Firenze 40, 20060, Trezzano Rosa (MI), Trezzano Rosa, Italy; 3 Department of Biomedical and Clinical Sciences, Unit of Obstetrics and Gynecology, ASST Fatebenefratelli Sacco University Hospital, University of Milan, Via Giovanni Battista Grassi 74, 20157, Milan, Italy

**Keywords:** probiotic, *Lactobacillus*, vaginal microbiota, community state types, *Gardnerella*, 16S rRNA gene profiling

## Abstract

Oral consumption of probiotics is practical and can be an effective solution to preserve vaginal eubiosis. Here, we studied the ability of orally administered *Lactobacillus paracasei* LPC-S01 (DSM 26760) to affect the composition of the vaginal microbiota and colonize the vaginal mucosa in nondiseased adult women. A total of 40 volunteers took oral probiotic (24 billion CFU) or placebo capsules daily for 4 weeks, and after a 4-week washout, they switched to placebo or probiotic capsules according to the crossover design. A total of 23 volunteers completed the study according to the protocol. Before and after capsule ingestion, vaginal swabs were collected for qPCR quantification to detect *L. paracasei* LPC-S01 and for 16S rRNA gene sequencing. Vaginal swabs were grouped according to their bacterial taxonomic structure into nine community state types (CSTs), four of which were dominated by lactobacilli. *Lactobacillus paracasei* LPC-S01 was detected in the vagina of two participants. Statistical modeling (including linear mixed-effects model analysis) demonstrated that daily intake of probiotic capsules reduced the relative abundance of *Gardnerella* spp. Quantitative PCR with *Gardnerella vaginalis* primers confirmed this result. Considering the pathogenic nature of *G. vaginalis*, these results suggest a potential positive effect of this probiotic capsule on the vaginal microbial ecosystem.

## Teaser

This study aims to assess whether a lactobacillus of vaginal origin can benefit the eubosis of the vaginal mucosa of childbearing age woman upon oral intake.

## INTRODUCTION

The human vagina represents a unique microbial ecosystem. Compared to that of other body sites, the vaginal microbiota is characterized by very low α-diversity (particularly in terms of evenness) and high intersubject variability (Zhou *et al*. 2013). In addition, the human vaginal environment is unique compared to that of other mammals, since it has a pH lower than 5 as a consequence of the abundant presence of a single acid-producing taxonomic bacterial group, i.e. lactobacilli (Miller *et al*. 2016). In particular, the vaginal microbiota of healthy women colonizes the approximately 1–4 ml of glycogen-rich fluid produced by the mucosa at a concentration of approximately 10^6^–10^8^ cells/ml (Danielsson, Teigen and Moi 2011). Specifically, the vaginal microbial ecosystem is nearly completely constituted by bacteria, 70% of which are most commonly represented by the homofermentative *Lactobacillus* species *Lactobacillus crispatus*, *Lactobacillus iners*, *Lactobacillus gasseri* and/or *Lactobacillus jensenii* (Ravel *et al*. 2011).

The vaginal mucosa and its microbial commensals interact closely, influencing different aspects of women's health, primarily fertility and susceptibility to infections (Smith and Ravel 2017). Specifically, autochthonous lactobacilli are believed to preserve vaginal health through various mechanisms, including the direct inhibition of pathogens associated with vaginosis (e.g. *Candida albicans*, *Gardnerella vaginalis, Escherichia coli, Leptotrichia* spp.*,  Mobiluncus* spp.*, Mycoplasma* spp.*, Peptostreptococcus* spp.*, Peptoniphilus* spp.*, Prevotella* spp.*, Sneathia* spp.*, Staphylococcus aureus and Streptococcus agalactiae*) through competitive exclusion for adhesion sites and nutrients, lactic acid production and bacteriocin secretion; the promotion of vaginal epithelial integrity (stimulating the secretion of mucin) and the modulation of immune responses (Younes *et al*. 2018).

The most common alterations in the composition of the vaginal microbiota involve a decrease in the presence of lactobacilli, excessive growth of a mixed microbial flora and increased microbial biodiversity (Onderdonk, Delaney and Fichorova 2016). This polymicrobial condition of vaginal mucosal dysbiosis, which is often associated with an abnormal odorous vaginal discharge, is associated with vulvovaginal conditions such as bacterial vaginosis (BV, also known as vaginal bacteriosis) and aerobic vaginitis. BV, although it may not be considered a disease in itself (Reid 2018), seems to promote pathological and infectious conditions. In fact, BV has been associated with an increased risk of contraction of sexually transmitted diseases, preterm birth and maternal or neonatal infections (van de Wijgert and Jespers 2017).

The maintenance of microbial homeostasis in the human vagina is therefore a fundamental condition for female health. In this context, the administration of probiotics, i.e. live microorganisms that can benefit health when administered in an adequate amount (Hill *et al*. 2014), is a practical and potentially effective strategy for preserving the eubiosis of the vaginal microbial ecosystem. Probiotics have been reported to be effective in the prevention and treatment of BV (Huang, Song and Zhao 2014) and urinary tract infections (Grin *et al*. 2013). Various probiotic products for benefiting vaginal health are available on the market and predominantly contain lactobacilli; some of these *Lactobacillus* strains have been isolated from the vaginal mucosa (e.g. *L. reuteri* RC-14); (Reid, Cook and Bruce 1987; Zhong *et al*. 1998) or from the urethra (e.g. *L. rhamnosus* GR-1); (Petrova *et al*. 2018) of healthy women and are administered in formulations for topical application in the form of tablets, ovules, capsules, gel or lavenders. Intriguingly, it has been suggested that oral administration may also have a positive influence on the vaginal microbial ecosystem (Macklaim *et al*. 2015). The idea of administering oral probiotic lactobacilli to benefit vaginal health originates from the hypothesis that these bacteria, which are adapted to colonize the mucous membrane of the vagina, can migrate from the gut and benefit vaginal health, similarly to several pathogens, which can ascend from the rectum and perineal skin to cause urogenital tract infections (Ahrné, Jeppsson and Molin 2005; Reid 2017). This hypothesis is supported by several studies reporting that orally administered lactobacilli can (i) migrate to the vagina (Reid *et al*. 2001a; Reid *et al*. 2001b; Reid *et al*. 2003; Morelli *et al*. 2004), (ii) reduce vaginal and rectal colonization by urogenital pathogens (Ho *et al*. 2016) and (iii) improve immune responses systemically through local (intestinal) immunomodulation (Lorea Baroja *et al*. 2007).

In the context described above, we show the results of an intervention study carried out by administering a vaginal *Lactobacillus* isolate *per os* to healthy women of reproductive age. We used a bacterial strain originally isolated from the vaginal mucosa of a healthy adult woman (*Lactobacillus paracasei* LPC-S01). This strain has been shown to possess characteristics compatible with its use as an intestinal probiotic, such as the ability to resist gastrointestinal transit *in vivo*, adhere to the intestinal epithelium *in vitro* and reduce NF-κB activation in the presence of inflammatory stimulation in polarized Caco-2 intestinal epithelial cells (Balzaretti *et al*. 2015). Such potential probiotic features of strain LPC-S01 may contribute to preventing intestinal dysfunctions, which have been associated with vaginal health (Reed *et al*. 2012; Drummond *et al*. 2016). The primary endpoint of this study was to assess the impact of LPC-S01 on the vaginal microbiota composition; in addition, we evaluated the ability of this strain to migrate to the vaginal mucosa once ingested.

## MATERIALS AND METHODS

### Intervention trial

**Figure 1.**
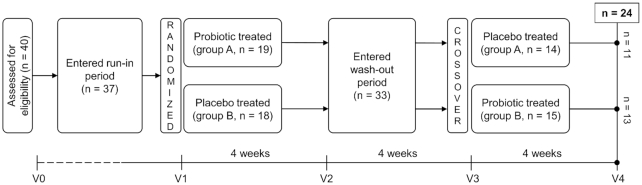
Diagram of study design and flow.

The VAG-LPC14 study was a randomized, double blind, placebo-controlled crossover trial (Fig. 1). The primary endpoint of the study was the evaluation of the ability of the probiotic strain to reach the vaginal environment and modify the composition of the vaginal microbiota. The study protocol was approved by the Research Ethics Committee of the Sacco Hospital, University of Milan (opinion no. 00  19288, 24/07/2015). A total of 40 healthy women of reproductive age (18 to 45 years) fulfilling all inclusion and exclusion criteria were randomized and entered the run-in phase according to the scheme shown in Fig. 1 (group A: probiotic first, n = 19, age ± standard deviation 30.4 ± 6.7 years; group B: placebo first, n=18, 30.5 ± 6.2 years). Each volunteer orally took one probiotic (24 billion CFU) or placebo capsule at least 10 min before breakfast every day for 4 weeks, and after a 4-week washout, they switched to placebo or probiotic capsules according to the crossover design. A 4-week interval between treatments was adopted, as it was shown to be sufficient for complete washout of probiotic cells from the gut in previous intervention trials carried out with an identical design (Ferrario *et al*. 2014; Gargari *et al*. 2016). Detailed information on the study participants and experimental protocol are provided in the Supplementary Methods.

### Probiotic and placebo capsules

The probiotic preparation (Pregyn^®^, Sofar S.p.A.) consisted of a gelatin capsule containing at least 24 billion viable cells of the bacterial strain *L. paracasei* LPC-S01 (deposited in the Deutsche Sammlung von Mikroorganismen und Zellkulturen (DSMZ) culture collection under the code DSM 2760; LPC-S01). The capsules contained silicon dioxide and magnesium stearate as antiagglomerants and were externally colored with titanium dioxide. The placebo and probiotic capsules were identical in color and shape. The placebo and probiotic capsules were directly provided in metal boxes with a plastic cap containing desiccant salts by the manufacturer of the Pregyn^®^ probiotic product (Sofar S.p.A.).

### Vaginal sample collection and total DNA extraction

Vaginal swabs were collected by the gynecologist (in the Department of Biomedical and Clinical Sciences, Unit of Obstetrics and Gynecology, ASST Fatebenefratelli Sacco University Hospital, University of Milan, Milan, Italy) following the standard vaginal swab collection procedure mentioned in the Manual of Procedures for Human Microbiome Project at visits V1, V2, V3 and V4 (according to the scheme in Fig. 1), transferred into 750 µl of PowerBead solution (Qiagen GmbH, Hilden, Germany) and stored at -80°C until they were processed for DNA extraction. In addition, during visits V1 to V4, the volunteers delivered a fecal sample collected during the previous 24 h in a special container that was provided during visit V0. The fecal specimens were also stored at -80°C until they were processed for DNA extraction, as follows. Vaginal swabs in PowerBead Solution were thawed on ice. Swabs were pressed several times by rotating on the inner wall of the sampling tube to release the entire amount of buffer or vaginal mucus adhered on the swab under aseptic conditions (later swabs were used for isolation of the target probiotic strain). Samples were then processed using a DNeasy® PowerLyzer® PowerSoil® Kit (Qiagen GmbH) with a minor modification consisting of sample incubation in PowerBead solution at 65°C for 10 min after the addition of C1 solution. Mechanical lysis of cells was carried out using a bead beater (Precellys 24, Bertin Technologies, Montigny le Bretonneux, France). A similar procedure was followed for DNA extraction from fecal samples. Specifically, after thawing on ice, samples were mixed vigorously for 2–3 min with a sterile spatula. Samples were weighed (250 mg) in a PowerBead tube, and DNA extraction was carried out as mentioned above. The DNA obtained from either vaginal or fecal samples was quantified in Take3 Micro-Volume plates analyzed in a microplate reader with Gen5 software (BioTek Instruments, Inc., Winooski, VT, USA), diluted to 10 ng/µl and stored at -80°C until use.

### *Quantification of* L. paracasei *LPC-S01 in vaginal and fecal samples through quantitative PCR*

The probiotic strain *L. paracasei* LPC-S01 was quantified in both vaginal and fecal samples by means of quantitative PCR (qPCR) with the strain-specific primers qS01a-F (5′-TGGAAGAGACCCTGCGAA-3′) and qS01a-R (5′-GAGGTTGATTCACAAACCGTGC-3′). These primers targeted a hypothetical protein coding sequence in the draft genome of strain LPC-S01 (Balzaretti *et al*. 2015) with an expected amplicon size of 181 bp. In addition, the total number of bacteria was quantified using the panbacterial primers 357F-907R, which targeted the V3-V5 region of the 16S rRNA gene (Muyzer, de Waal and Uitterlinden 1993). qPCR amplification was carried out in a final volume of 15 μl containing 7.5 µl of EvaGreen® Supermix (Bio-Rad Laboratories S.r.l., Segrate, Italy), 0.5 μM each primer and 50 ng of metagenomic DNA from vaginal or fecal samples. Amplification was carried out using the following thermal cycling conditions: an initial hold at 95°C for 3 min and 39 cycles at 95°C for 30 s, 58°C for 30 s and 72°C for 30 s. Melting curves were analyzed with Bio-Rad CFX Manager 3.1 to confirm the specificity of the amplification products. A standard calibration curve for quantification of LPC-S01 cells was prepared by mixing different numbers of LPC-S01 cells from vaginal swabs. For this purpose, several vaginal swab samples were collected from the volunteers and transferred into 750 µl of PowerBead solution. The swab samples were combined in one tube, mixed vigorously and equally distributed into seven tubes. After quantification of *L. paracasei* LPC-S01 in a C6 Plus BD Accuri™ flow cytometer (BD Biosciences, San Jose, CA, USA), probiotic cells were added to each swab sample (from 10^6^ to 10^1^ cells), except for one sample used as a control without the addition of bacterial cells; then, DNA was extracted as described above. Similarly, a standard calibration curve for quantification of the probiotic strain in fecal samples was prepared as previously described (Arioli *et al*. 2018). In addition, the standard calibration curve for quantification of the total bacterial cell count in vaginal swab samples was prepared by creating a vaginal swab pool. For this purpose, vaginal swab samples were provided by volunteers in 750 µl of PowerBead solution and pooled. Then, the vaginal swab pool was mixed properly and concentrated by centrifugation. After determination of the total bacterial cell concentration by flow cytometry, the concentrated swab pool was serially diluted, and each dilution was subjected to DNA extraction following the abovementioned procedures. Then, DNA obtained from these different dilutions of the vaginal swab pool was used for quantitative PCR to generate a standard calibration curve for estimation of the total bacterial cell count in vaginal swab samples collected in the study.

### *Quantification of* G. vaginalis *in vaginal samples through qPCR*

The abundance of *G. vaginalis* in vaginal swab samples was quantified by qPCR with the species-specific primers Gard_LdhF, 5′-GTTATTACTGCTGGTGCTCG-3′ and Gard_LdhR, 5′-GCTCGCCAGCAATATAAGCG-3′, which targeted the lactate dehydrogenase gene, with an expected amplicon size of 301 bp according to the protocol provided in the supplementary material.

### *Isolation of* L. paracasei *LPC-S01 from vaginal swabs*

After aseptic processing for total DNA extraction, vaginal swabs were transferred into de Man-Rogosa-Sharpe (MRS) broth (pH 6.5) supplemented with ribose (1% w/v), vancomycin (1 µg/ml) and kanamycin (10 µg/ml) (rvkMRS) for semiselective isolation of the probiotic strain. The swab-inoculated broth tubes were incubated at 37°C for 48 h under anaerobic conditions (Anaerocult A, Merck KGaA, Darmstadt, Germany). The broth cultures were used for DNA extraction and qPCR with LPC-S01-specific primers as described above. Simultaneously, each culture broth was spread on agar medium (rvkMRS agar). Randomly selected colonies were examined under a microscope to determine their morphology and were identified by colony PCR (Arioli *et al*. 2018).

### 16S rRNA gene profiling of vaginal samples

To establish whether oral administration of the vaginal bacterium *L. paracasei* LPC-S01 modified the composition of the vaginal microbiota, we used 16S rRNA gene profiling to analyze four vaginal swabs per subject collected from women who completed the intervention study according to the protocol (n = 24). Specifically, the vaginal bacterial community was characterized immediately before and after the probiotic and placebo phases, according to the crossover scheme shown in Fig. 1. DNA extracted from vaginal swabs was analyzed at the Institute for Genome Sciences (University of Maryland, School of Medicine, Baltimore, MD, USA) through 16S rRNA gene profiling with Illumina HiSeq 2500 rapid run sequencing of the V3-V4 variable region (forward primer, 5′-ACTCCTRCGGGAGGCAGCAG-3′; reverse primer, 5′-GGACTACHVHHHTWTCTAAT-3′), which was performed using a two step-PCR protocol as described in detail in (Elovitz *et al*. 2019). The 16S rRNA gene profiling data for the analysis of vaginal microbiota were analyzed using R statistical software (version 3.1.2) with the DADA2 software package (Callahan *et al*. 2016) associated with the taxonomic assignment tool speciateIT. For the V3-V4 region, DADA2 used reads truncated after nt 255 for forward reads and at nt 225 for reverse reads. Taxonomic assignment was performed with the SILVA database according to a custom pipeline freely available on GitHub (https://github.com/Ravel-Laboratory/speciateIT). α-Diversity (Chao1, Simpson, invSimpson and Shannon) and β-diversity index analyses were performed using R software to describe the intra- and intersubject diversity, respectively. Stratification of the vaginal microbiota into community state types (CSTs) was carried out on the basis of the relative abundance of the most represented taxon in the sample, using 50% of total reads per sample as the cutoff. If no taxon in a sample exceeded an abundance of 50%, that sample was assigned to CST IV. Metadata have been deposited in the European Nucleotide Archive of the European Bioinformatics Institute under accession code PRJEB30833.

### Statistical analysis

Statistically significant changes in the relative abundances of bacterial taxa were determined through the Wilcoxon signed rank test for paired data using the Benjamini-Hochberg correction (Haynes 2013) when needed. Correlation analyses with the relative abundances of vaginal taxa were performed using the Kendall and Spearman formulas. Significance was set at P ≤ 0.05; significance in the range of 0.05 < *P*< 0.10 was accepted as a trend. To find associations among the probiotic treatment and changes in bacterial relative abundances, a machine learning supervised linear mixed model (LMM) algorithm was adopted using the ‘lmer’ function in the ‘lme4’ library (Bates *et al*. 2015) in R statistical software (version 3.4.2). LMM fit was tested with the Akaike information criterion. The nonparametric *analysis of similarities* statistical test (ANOSIM) was used to infer significant differences among CST groups.

## RESULTS

### Baseline composition of the vaginal microbiota of the volunteers

We analyzed the vaginal microbiota of the 37 healthy (nondiseased) volunteers who entered the run-in phase (Fig. 1). For this purpose, 16S rRNA gene profiling was performed on a vaginal swab collected from each subject at the beginning of the intervention (baseline; time point V1 in Fig. 1). The number of total reads generated was 6,470,846 for the 109 total vaginal samples, with an average of 30 097 reads per sample. The number of filtered total reads was 5,501,139, and of these, 5,099,077 reads had been merged. In particular, the number of merged reads per vaginal sample was 13,340± 8,677 (mean ± standard deviation) (range, 4,570–11,594). Following taxonomic assignment of the sequencing reads, we found a total of 69 bacterial taxa (mean of 17 and median of 15 taxa per sample). No taxon was found in all subjects. The most dominant bacteria were *L. crispatus* and *L. iners*, each detected in 68% of subjects, followed by members of the genera *Finegoldia* (65%), *Streptococcus* (65%) and *Bifidobacterium* (62%). All other taxa were found in less than 50% of the subjects. In particular, further taxonomic analysis of the reads assigned to *Bifidobacterium* carried out by manual screening of GenBank (database of 16S ribosomal RNA sequences) through BLASTn suggested that most of these reads corresponded to the species *B. breve* (79% of the *Bifidobacterium* spp. reads) and *B. longum* (21%) (not shown).

Analysis of the vaginal microbiota taxonomic composition at baseline (V1) allowed stratification of samples into eight distinct types of bacterial communities, named CSTs, that differed in both the composition and relative abundance of taxa (Ravel *et al*. 2011) (CSTs; Fig. S1, Supporting Information). Five of these CSTs (from I to V) resembled the CSTs originally described for the human vaginal microbial ecosystem (Ravel *et al*. 2011). In addition, we found three more CSTs dominated by members of the genera *Bifidobacterium*, *Streptococcus* and *Alloscardovia*. The most prevalent CSTs were I (*L. crispatus*-dominant; found in 32% of subjects; n = 12), III (*L. iners*-dominant; 24%; n = 9) and IV (mixed community; 14%; n = 5). In contrast to the other CSTs, CST IV was characterized by the absence of a dominant taxon (i.e. one accounting for more than 50% of the reads), as also evidenced by the inverse Simpson α-diversity index, used as a measure of taxonomic evenness (Fig. S1, Supporting Information).

**Figure 2.**
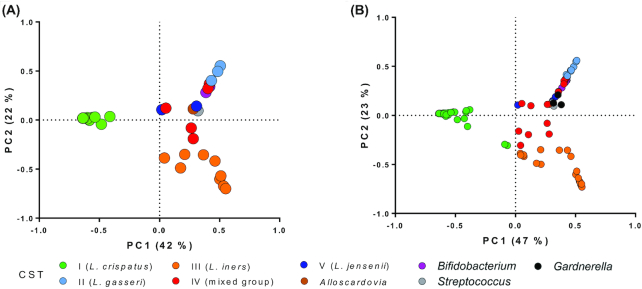
PCA plots obtained using the relative abundances of bacterial taxa in each vaginal swab. Each point in the plots indicates a single vaginal swab. The color of each dot in the plots indicates the CST of the sample according to the legend reported under the chart; the colors correspond to the colors used in Fig. 3. **A**, baseline samples (n = 37, i.e. vaginal samples collected at V1 from the 24 volunteers who completed the study and 13 who dropped out); **B**, all samples (n = 109, i.e. the 37 samples collected at V1 and the samples collected at V2, V3 and V4 from the 24 volunteers who completed the study).

Principal component analysis (PCA) was performed using the relative abundances of bacterial taxa in each subject at baseline (V1) to visualize the relationships among vaginal bacterial communities (Fig. 2A). In the resulting two-dimensional PCA loading plot, which explained 70% of the diversity, the *L. crispatus*-dominant (I) and *L. iners-*dominant (III) CSTs clustered separately from the other samples (ANOSIM r = 0.98, *P* < 0.01; Fig. 2A).

### *Impact of oral administration of* L. paracasei *LPC-S01 on the vaginal microbiota composition*

**Figure 3.**
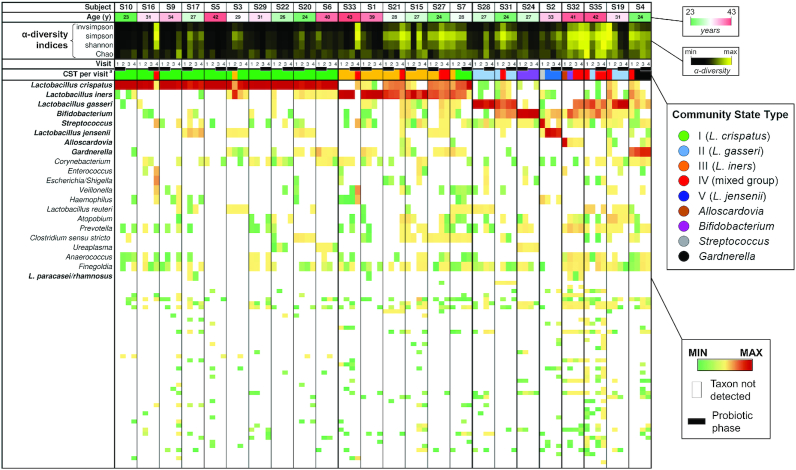
Composition of the vaginal microbiota of the 24 reproductive-age women who completed the VAG-LPC14 study (see Fig. 1 for details on the study design). The green-white-red heatmap indicates the ages of volunteers; the age in years is indicated in each box. The black-yellow heatmap represents the α-diversity indices, where the color indicates the values from minimum (black) to maximum for each respective index. The green-yellow-red heatmap represents the log_10_-transformed relative abundances of microbial taxa found in the vaginal bacterial communities. *a*, CST observed for vaginal samples at each visit; each color corresponds to a specific CST according to the legend reported on the right. Subjects are clustered according to the CST observed at visit 1.

We also used PCA as described above to visualize the vaginal bacterial communities of all vaginal swab samples collected during the study (Fig. 2B). This analysis confirmed the distribution observed using the baseline samples, with bacterial communities assigned to CSTs I and III that clustered separately from the others (ANOSIM r = 0.97, P < 0.01; Fig. 2B). In general, the CSTs were mostly stable; in fact, of 72 possible transitions (24 subjects per 3 phases, i.e. probiotic, washout, and placebo), the CST changed 16 times (21%): 7 times during the placebo phase, 5 times during the washout and 4 times during the probiotic administration phase (Fig. 3). In particular, CST-I was the most stable: of 30 possible transitions (n = 10), this CST changed only twice (Fig. 3).

**Figure 4.**
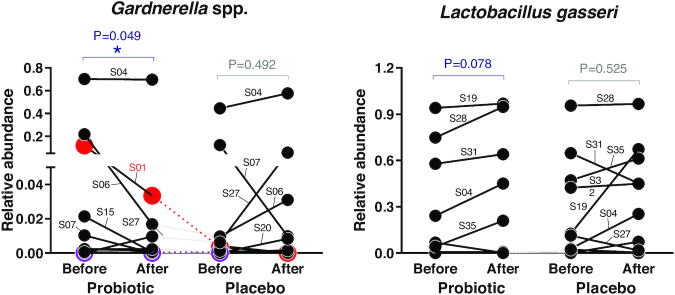
Relative abundance of vaginal bacterial taxa that were significantly modified during the interventions (n = 24). Volunteers who tested positive for the presence of the probiotic strain LPC-S01 in a vaginal swab are represented in red (S01) and violet (S17). The subject code is indicated for higher-abundance samples. Statistically significant differences were determined according to the Wilcoxon signed rank test. The dotted lines indicate the subjects randomly selected to receive the probiotic first.

Subsequently, to identify whether probiotic intake modified the relative abundance of specific taxa, we performed statistical analysis with the Wilcoxon signed rank test for taxa that were present in at least 30% of the samples (arbitrary limit). We did not find any significant modification of bacterial taxa upon intervention with placebo; in contrast, after probiotic intake, we found a significant decrease in the genus *Gardnerella* (*P* = 0.049; Fig. 4) and an increasing trend for the species *L. gasseri* (*P* = 0.078; Fig. 4). Subsequently, bacterial relative abundance data were also analyzed by means of LMM analysis to observe the predictive power for the changes in the bacterial community due to the treatment. LMM analysis revealed a significant association with treatment only for the genus *Sutterella* (*P* = 0.035) and a trend toward significance for *Gardnerella* (*P* = 0.080) and *L. crispatus* (*P* = 0.085). A significant reduction in the abundance of *Gardnerella* over the course of probiotic intervention was also observed by qPCR, which was performed using *G. vaginalis*-specific primers on DNA extracted from vaginal swabs (Fig. S2, Supporting Information).

### Tolerability and safety of the intervention

Two subjects, viz., S17 and S18, experienced at least one adverse event while being treated with the placebo or Pregyn^®^, respectively. No serious adverse events occurred. Table S1 (Supporting Information) summarizes the adverse events that occurred during the trial. Most of the events suspected to be related to the treatment were gastrointestinal disorders (abdominal pain, constipation, diarrhea, dyspepsia, flatulence, meteorism and nausea), and no significant difference between treatment groups was detected. The total number of adverse events and the number of events per subject did not differ between the two treatments, nor did the number of adverse events suspected to be related to the treatment.

### Effect of probiotic treatment on the predicted metabolic potential of the vaginal microbiota

The 16S rRNA amplicon sequencing data set was also used to infer the functional contribution of the vaginal bacterial communities by means of the PICRUSt computational approach. Analysis of the obtained functional gene count matrix revealed that the relative levels of 34 putative genes changed during the placebo phase (32 genes increased and 2 decreased), whereas only 10 potential bacterial genes were significantly modified (9 increased and 1 decreased) during the probiotic phase. The results of a BLASTP search (not shown) showed that most of the predicted genes that increased in the placebo phase (n = 21) but did not change during the probiotic intervention phase, were not present in the genome of homofermentative lactobacilli (e.g. *L. crispatus*, *L. iners*, *L. gasseri* and *L. jensenii*). These genes included eight predicted genes involved in the tricarboxylic acid cycle and the genes encoding indolepyruvate ferredoxin oxidoreductase, the type IV pilus assembly protein PilC and glutathionylspermidine synthase (Fig. S3, Supporting Information). In addition, the only predicted genes with reduced levels in the placebo phase are putatively involved in the synthesis of nicotinate and nicotinamide. In contrast, half of the predicted genes with increased levels during the probiotic phase (n = 5) putatively encode membrane transport proteins.

### *Recovery of strain* L. paracasei *LPC-S01 from the vaginas of volunteers*

**Figure 5.**
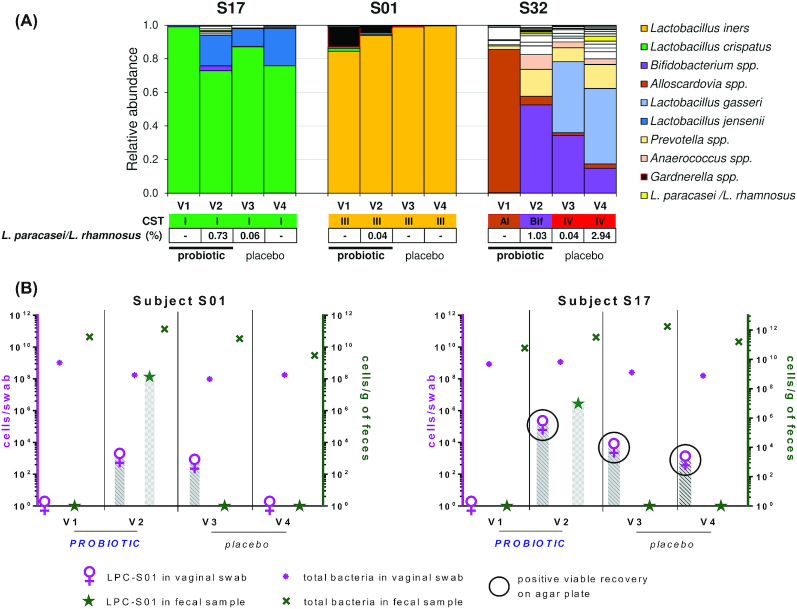
Recovery of *L. paracasei* LPC-S01 in vaginal swabs. **A**, histograms indicating the bacterial composition of vaginal swabs as determined by 16S rRNA gene profiling; only volunteers who tested positive for the presence of sequencing reads assigned to the taxonomic group *L. paracasei*/*L. rhamnosus* are shown. **B**, qPCR quantification of *L. paracasei* LPC-S01 (with strain-specific primers) and total bacteria (with pan-bacterial primers) in fecal and vaginal samples. Samples from which LPC-S01 were isolated on agar plates are shown in the black circle. Only those subjects whose vaginal swabs were positive for LPC-S01 are shown.

16S rRNA gene profiling of vaginal swabs revealed the presence of reads taxonomically assigned to *L. paracasei*/*L. rhamnosus* group in 3 subjects (S1, S17, and S32; Fig. 5A). In particular, *L. paracasei*/*L. rhamnosus* reads appeared in these three volunteers after probiotic intervention (Fig. 5A); therefore, we hypothesized that these results might have been a consequence of the migration of *L. paracasei* LPC-S01 from the gut to the vaginal mucosa. To test this hypothesis, we performed qPCR analyses with LPC-S01-specific primers using DNA isolated from all vaginal swabs collected during the study. According to 16S rRNA gene profiling, significant amplification was obtained only for subjects S1 (for the swab collected at V2) and S17 (at V2 and V3), not for S32 (Fig. 5B). In addition, we were able to isolate LPC-S01 on rvkMRS agar plates only from the swabs collected from subject S17 at V2, V3 and V4 (Fig. 5B).

Strain-specific qPCR experiments were also carried out to quantify *L. paracasei* LPC-S01 in fecal samples. LPC-S01 was detected exclusively in fecal samples collected at the end of the probiotic intake period for 21 of 23 investigated volunteers at concentrations ranging between 5.6 and 8.1 log_10_ cells per gram of feces (Fig. S4).

### Correlations among vaginal bacterial taxa

Finally, we performed correlation analyses to assess the potential associations among the different bacterial taxa within the vaginal microbiota. To this end, Spearman and Kendall tests were performed considering the four sets of data obtained over the course of the intervention (i.e. before and after the probiotic and before and after the placebo intake phases) and with the median data from four samples per subject. Each profiling data set obtained provides information at a single time point for each volunteer and may have limitations connected to the variability of the microbiota over time; on the other hand, the analysis of median profiling data may include error that depends on the influence of the probiotic intervention. Therefore, we combined the results of the different analyses to compensate for limitations and identify more plausibly valid (stable) correlations.

**Figure 6.**
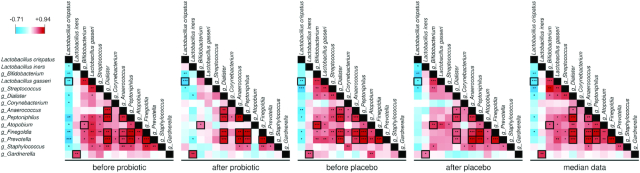
Correlations among the relative abundances of vaginal bacterial taxa calculated at each of the four phases of the trial (i.e. before and after the probiotic and the placebo treatments; n = 24). In addition, correlations with median data at the four time points for each volunteer are shown. The squares with bold black margins highlight the correlations maintained in all five correlation analyses. Only vaginal taxa found in at least 30% of vaginal samples were considered in the analysis. This figure only includes taxa whose abundance significantly correlated with at least one vaginal taxon according to Kendall's tau rank correlation. The colors in the heatmap represent the R-value obtained by Spearman correlation analysis. The asterisks indicate the Kendall's tau rank correlation: *, *P* < 0.05; **, *P* < 0.01; ***, *P* < 0.001.

These analyses showed that significant correlations among vaginal microbial taxa decreased from 47 to 22 and from 43 to 29 during the probiotic and placebo interventions, respectively, whereas the median profiling data revealed 38 significant correlations (Fig. 6). Thirteen correlations were found in all five analyses (Fig. 6). Specifically, we consistently found a significant positive association among the five vaginal commensal genera *Dialister*, *Anaerococcus*, *Peptoniphilus*, *Finegoldia* and *Prevotella*; in addition, we observed a positive association between the *Actinobacteria* genera *Bifidobacterium* and *Atopobium*. In all analyses, *L. crispatus* was negatively associated with *L. gasseri*, whereas *L. iners* was positively correlated with the genus *Gardnerella*.

Overall, these results suggest that the presence and abundance of specific vaginal bacterial taxa are mutually associated.

## DISCUSSION

To establish the effect of the administration of the *L. paracasei* LPC-S01 strain on the composition of the vaginal microbiota, a crossover study design was adopted. This choice was made to address the high intersubject diversity that reportedly characterizes the microbial ecosystem of the human vagina (Zhou *et al*. 2013). Characterization of the volunteers at baseline confirmed the expected high β-diversity of the vaginal microbiota, highlighting a clear stratification in CSTs for this microbial ecosystem (Fig. 2; Ravel *et al*. 2011). Of the eight CSTs identified in this study, five corresponded to those originally described by Ravel *et al*. 2011. In addition, subjects whose vaginal mucosa was dominated by other bacterial genera, i.e. *Alloscardovia*, *Streptococcus* and *Bifidobacterium*, were identified. The first two genera could characterize transitional states, as suggested by the fact that these community states were not identified in any other analyses of these or other volunteers. In contrast, the CST dominated by the genus *Bifidobacterium*, which was identified in four subjects at baseline, was shown to be constant throughout the study for the only subject among these four (S24) who completed the trial according to the protocol (Fig. S1 (Supporting Information) and Fig. 3). The presence of *Bifidobacterium* spp. as dominant members of the vaginal microbiota of some healthy reproductive-age women has already been reported (Hyman *et al*. 2005; Albert *et al*. 2015). In particular, Freitas and Hill (Freitas and Hill 2017) showed that bifidobacterial strains isolated from healthy vaginal mucosae produce lactic acid at levels comparable to those produced by *L. crispatus* (the species most commonly associated with a healthy vaginal microbial ecosystem) and may tolerate the low pH of the vaginal environment well. On this basis, Freitas and Hill proposed that bifidobacteria may be as protective as lactobacilli. Similarly, Campisciano *et al*. proposed that bifidobacteria (particularly *B. breve*) may counteract the poor colonization of the vaginal mucosa by lactobacilli, preserving vaginal health primarily through the production of lactic acid (Campisciano *et al*. 2018). The most abundant end product of *Bifidobacterium* catabolism is acetic acid (theoretically, 1.5 moles of acetate and 1 mole of lactate are produced from 1 mole of glucose), which may lower the pH as efficiently as lactic acid and is known to possess antimicrobial activity. It can therefore be speculated that, in addition to lactic acid, acetic acid produced *in situ* by bifidobacteria may contribute to the maintenance of vaginal health.

The impact of probiotics on the vaginal microbiota has often been investigated under physiological conditions characterized by an altered vaginal microbial ecosystem, such as BV (Bodean *et al*. 2013), aerobic vaginitis (Heczko *et al*. 2015) and other vaginal infections (Vujic *et al*. 2013), or after antibiotic therapy (Martinez *et al*. 2009). Among probiotic products, those intended for intravaginal administration have been most commonly investigated (Bisanz *et al*. 2014; Kovachev and Vatcheva-Dobrevska 2015; Verdenelli *et al*. 2016). In contrast, intervention studies with oral probiotics involving healthy (nondiseased) populations are limited. For instance, administration of a multispecies probiotic product for 4 weeks was shown to limit the decrease in bifidobacteria and the increase in *Atopobium* spp. occurring in the vagina of women during late pregnancy, as determined through qPCR analysis (Vitali *et al*. 2012). On the other hand, Gille *et al*. reported that the effect of an 8-week oral administration of the human urogenital strains *L. rhamnosus* GR-1 and *L. reuteri* RC-14 (10^9^ colony-forming units) to women with < 12 completed weeks of pregnancy did not differ from that of placebo on modifying the vaginal microbiota composition, as assessed through microscopic observation (Nugent scoring) (Gille *et al*. 2016). However, in two other placebo-controlled trials, Nugent scoring and cultivation experiments revealed that oral administration of the same strains significantly increased the level of vaginal lactobacilli (Reid *et al*. 2001a; Reid *et al*. 2003), suggesting 10^8^ viable bacterial cells per day as the required minimal dose (Reid *et al*. 2001a). An increase in vaginal *Lactobacillus* spp. was also reported upon oral intake of the commercial probiotic strains *L. acidophilus* La-14 and *L. rhamnosus* HN001 in combination with bovine lactoferrin, as assessed through qPCR (De Alberti *et al*. 2015). In general, the assessment of vaginal microbiota modifications induced by probiotic administration in healthy women is made difficult by the marked stratification of human vaginal bacterial communities (Zhou *et al*. 2013). In our study, the probiotic intervention did not appear to affect the stability of the vaginal CSTs; however, when we considered specific bacterial taxa, we observed a significant reduction in the reads ascribed to the genus *Gardnerella*. *Gardnerella* is a genus in the family *Bifidobacteriaceae* that—to date—includes only the species *G. vaginalis* (according to NCBI Taxonomy, https://www.ncbi.nlm.nih.gov/taxonomy; Taxonomy ID: 2701). This anaerobic bacterium commonly exists as a minority member of the normal (nondysbiotic) vaginal microbiota of most women (prevalence of approximately 85%; Janulaitiene *et al*. 2017), acting as a pathobiont; in fact, an increase in the relative abundance of *G. vaginalis* leads to BV and is consequently associated with preterm birth and an increased risk of acquiring sexually transmitted infections (Kairys and Garg 2018). BV is a dismicrobism that is characterized by concomitant expansion of several other vaginal pathobionts, such as *Atopobium*, *Fusobacterium*, *Mobiluncus*, *Peptoniphilus* and *Prevotella*, in addition to *G. vaginalis*. However, *G. vaginalis* plays a dominant role in the onset of this dysbiotic polymicrobial consortium because it can efficiently adhere to the vaginal epithelium and compete with lactobacilli (Machado, Jefferson and Cerca 2013), initiating the establishment of the multibacterial biofilm community that characterizes BV (Schwebke, Muzny and Josey 2014; Machado and Cerca 2015). Therefore, although *G. vaginalis* includes both virulent and avirulent strains, the observed reduction in *Gardnerella* abundance in the vaginal microbial ecosystem induced by oral intake of *L. paracasei* LPC-S01 may plausibly favor the maintenance of vaginal eubiosis.

The high intersubject taxonomic variability of the vaginal microbial environment, together with the relatively low number of volunteers considered in this study, limited the possibility of finding specific bacterial taxa that were significantly modified by the probiotic intervention. However, despite their high taxonomic variability, microbial communities reportedly possess a stable functional structure (Louca *et al*. 2016; Gibbons 2017); therefore, we also studied the metabolic potential of the vaginal microbiota predicted via the PICRUSt bioinformatic tool. This analysis provided four major results: the predicted genes whose relative abundance was significantly modified during the placebo phase (i) were approximately three times greater in number than the modified genes identified for the probiotic treatment (34 genes vs 10 genes, respectively), (ii) were mostly increased in relative level after the intervention (only 2 decreased), (iii) putatively belonged to the genomes of aerobic and/or facultative anaerobic bacteria and (iv) were not present in the genome of the most common commensal lactobacilli of the human vaginal mucosa. Considering that the human vaginal microbiota is characterized by high temporal instability (Zhou *et al*. 2013), possibly deriving, for instance, from menstruation and sexual activity (Eschenbach *et al*. 2000; Mitchell *et al*. 2012), our results on the metabolic potential suggest that the probiotic intervention may have limited the transition of the vaginal microbiota toward a lactobacilli-reduced multimicrobial community structure that is potentially linked to dysbiotic states and may facilitate the colonization and expansion of *Gardnerella*.

Our data confirmed that CSTs dominated by *L. crispatus* (CST-I) and *L. iners* (CST-III) were the most common CSTs among nondiseased women of reproductive age (Ravel *et al*. 2011). In particular, we observed that CST-I, which is generally recognized as the most protective against the acquisition of sexually transmitted diseases (Lewis, Bernstein and Aral 2017), displayed high stability throughout the study. In contrast, CST-III, dominated by *L. iners*, demonstrated transitions to a mixed CST (CST IV) in three of the six subjects belonging to this group. Accordingly, the *L. iners*-dominated vaginal compositional state was shown to be more commonly transitional toward dysbiosis than were the other states. *L. iners* has been associated with preterm delivery (Petricevic *et al*. 2014) and increased susceptibility to *Chlamydia trachomatis* infection (van Houdt *et al*. 2018). These observations make it difficult to establish whether *L. iners* is a ‘friend or foe’ in the vaginal mucosa (Petrova *et al*. 2017). However, the potential pathobiontic attitude of *L. iners* may be explained by some physiological and genetic features of this bacterium, including the production of less lactic acid than *L. crispatus* and only in the ‘levo’ optical form (Witkin *et al*. 2013). In addition, *L. iners* has been shown to significantly induce pattern recognition receptor signaling, resulting in proinflammatory modulation of the innate immune response (Doerflinger, Throop and Herbst-Kralovetz 2014). Moreover, *L. iners* may secrete a cholesterol-dependent pore-forming toxin named inerolysin, which shares similarity with vaginolysin, the main virulence factor of *G. vaginalis* (Rampersaud *et al*. 2011). The frequent isolation of *L. iners* during the transition between BV and non-BV states (Jakobsson and Forsum 2007) is apparently consistent with the results of our correlation analyses, which revealed a significant positive association between *L. iners* and *Gardnerella* at all studied time points (Fig. 6).

Correlation analyses showed that the bacterial genera *Finegoldia*, *Peptoniphilus*, *Anaerococcus*, *Dialister* and *Prevotella*, which are generally present at high levels in BV, were mutually associated throughout the entire study. *Peptoniphilus*, *Anaerococcus* and *Finegoldia* are Gram-positive anaerobic cocci belonging to the *Firmicutes* class *Tissierella*; these bacteria are part of the commensal microbiota of several body sites, where they can become opportunistic pathogens, as demonstrated by their frequent isolation from clinical specimens, such as blood, and from abscesses and skin, joint, bone, respiratory tract and urogenital tract infections (Murdoch 1998; Shilnikova and Dmitrieva 2015). *Dialister* is an anaerobic *Firmicutes* genus of the class *Negativicutes*; similar to the other three genera mentioned above, *Dialister* includes opportunistic bacteria with pathogenic potential that have been isolated from various infections, including periodontitis (Contreras *et al*. 2000), endodontic infections (Rolph *et al*. 2001), brain abscesses (Rousee *et al*. 2002), and bacteremia (Pierre Lepargneur, Dubreuil and Joseph 2006). Finally, the Gram-negative genus *Prevotella*, belonging to the phylum *Bacteroidetes*, is known to disrupt innate immune responses in the vaginal epithelium (Doerflinger, Throop and Herbst-Kralovetz 2014) and was shown to be the only discriminative bacterium present under several conditions, including BV, obesity and papillomavirus infection, in a Korean twin-family cohort (Sung *et al*. 2006; Si *et al*. 2017). These five genera of opportunistic bacteria, which are more abundant in CST IV, might plausibly proliferate in the vaginal mucosa when the presence of lactobacilli, especially *L. crispatus*, decreases. Accordingly, in our study, *L. crispatus* was the only vaginal taxon that showed negative associations with several other vaginal taxa, including BV-associated bacteria. These results reinforce the idea that *L. crispatus* is the most efficacious vaginal commensal in maintaining the microbial homeostasis of the human vagina.

Orally administered probiotics, similar to intestinal bacteria, have been hypothesized to migrate to the vagina from the intestine (Reid *et al*. 2001b). Reisolation of probiotics from the vaginal mucosa upon oral ingestion was first reported by G. Reid *et al*. for the strains *L. rhamnosus* GR-1 and *L. fermentum* RC-14 (Reid *et al*. 2001b). However, very few other studies have investigated the vaginal recovery of orally administered probiotics (Ahrné, Jeppsson and Molin 2005). In our study, the bacterial strain investigated, i.e. the vaginal isolate *L. paracasei* LPC-S01, was detected in vaginal swabs after oral administration in at least 2 of 24 subjects who completed the study according to the protocol. The frequency of vaginal recovery of this probiotic strain was therefore low; however, these results confirm that colonization of the vaginal mucosa by orally ingested *Lactobacillus* spp. may be a possible event. Probiotic migration into the vaginal mucosa might have been limited by possible variations in the ability of LPC-S01 to colonize the gastrointestinal tract. In support of this hypothesis, the highest concentration of LPC-S01 in feces was found for subject S01, who was one of the two volunteers positive for LPC-S01. However, there were too few samples to draw any conclusions.

In conclusion, this study confirms that the vaginal microbiota of nondiseased Caucasian women of reproductive age from North Italy can be stratified into the same community structures previously described for other populations. In addition, the *L. crispatus*-dominated CST was confirmed to be the most common among white reproductive-age women, followed by the *L. iners*-dominated CST. Finally, this study shows that the vaginal bacterial ecosystem of healthy women is quite stable; however, oral administration of LPC-S01 may influence the relative abundance of specific taxa such as *G. vaginalis*. This finding suggests a potential positive effect of this probiotic capsule on the vaginal microbial ecosystem. To confirm this preliminary result, further trials must be carried out, preferentially focusing on women with an intermediate or polymicrobial (i.e. CST IV) vaginal microbiota.

## Supplementary Material

fiaa084_Supplemental_File
