# Supplementary material for: Effect of oral consumption of capsules containing Lactobacillus paracasei LPC-S01 on the vaginal microbiota of healthy adult women: a randomized, placebo-controlled, double-blind crossover study
Source: FEMS Microbiol Ecol. 2020 May 8;96(6):fiaa084. doi: 10.1093/femsec/fiaa084 (PMC7261233; doi:10.1093/femsec/fiaa084)
Supplement: fiaa084_Supplemental_File [file fiaa084_supplemental_file.docx]

**Effect of oral consumption of capsules containing *Lactobacillus paracasei* LPC-S01 on the vaginal microbiota of healthy adult women: a randomized, placebo-controlled, double-blind crossover study**

Ranjan Koirala^1§^, Giorgio Gargari^1§^, Stefania Arioli^1^, Valentina Taverniti^1^, Walter Fiore^2^, Elena Grossi^3^, Gaia Maria Anelli^3^, Irene Cetin^3^, Simone Guglielmetti^1^*

^1^Division of Food Microbiology and Bioprocesses, Department of Food, Environmental and Nutritional Sciences, University of Milan, Milan, Italy.

^2^Sofar S.p.A., Trezzano Rosa, Italy.

^3^Department of Biomedical and Clinical Sciences, Unit of Obstetrics and Gynecology, ASST Fatebenefratelli Sacco University Hospital, University of Milan, Milan, Italy.

* Address correspondence to Simone Guglielmetti, [simone.guglielmetti@unimi.it](mailto:simone.guglielmetti@unimi.it).

^§^ Both authors contributed equally to this work.

**Supplementary methods**

*Study participants*

Forty nondiseased (healthy) women were assessed for participating in the VAG-LPC 14 [“Evaluation of the effect of PREGYN capsules (containing *Lactobacillus paracasei* LPC-S01) consumption on the vaginal microbiota composition of healthy adult women: a randomized, placebo-controlled, double-blind cross-over study”] study at U.O.C. Obstetrics and Gynecology of Sacco Hospital in Milan (**Fig. 1**). Thirty-seven women (aged 34.9 ± 10.7 y; BMI: 22.5 ± 2.7 kg/m^2^) entered the run-in period and were randomized into groups receiving the probiotic or placebo according to the crossover design shown in Fig. 1. The eligibility criteria were good general health, age between 18 and 45 y, and a signed informed consent form. The exclusion criteria were as follows: urogenital infections in the previous 3 months (especially bacterial vaginosis); urogenital abnormalities; ascertained or planned pregnancy or breastfeeding; intake of antibiotics and/or probiotics in the month before the start of the trial; systemic or topical therapy with steroids, glucocorticoids with rapid hepatic passage such as beclometasone dipropionate or budesonide, in progress or within the month prior to enrollment; chronic intestinal inflammatory diseases; intestinal diseases of infectious, actinic, endocrine or drug origin (microscopic colitis); immunodeficiency; malignant neoplasms of any kind, or a history of previous malignancy (history of other malignant neoplasms that were surgically removed without recurrence within the five years prior to entry into the study were still eligible); any renal, hepatic, hematological, cardiovascular, pulmonary, neurological, psychiatric, immunological, gastrointestinal or endocrine disease, if clinically significant; any severe pathology that could interfere with the treatment; recent history or suspicion of alcohol or drug abuse; presence of dementia of any kind or other possible causes of progressive deterioration of the ability to understand or any psycho-physical disability that reduces the ability to take the prescribed therapy as planned; inadequate reliability or the presence of conditions that could lead to a lack of voluntary compliance with the protocol; or previous participation in this study.

*Treatment and study scheme*

Each volunteer was initially trained on the entire procedure to be followed, which consisted of a total of 5 visits for each volunteer, each to be performed 14 days after the end of menstruation by the subject. During the first visit (V0), the informed consent and personal data of the volunteer were collected. In addition, the volunteer received general information about dietary changes to be followed in the following 4 weeks of the run-in period. Specifically, the volunteers were asked to follow their conventional diet with the intake of probiotic fermented milks, probiotic food supplements, and prebiotic food supplements prohibited. At the end of this run-in, the volunteers were randomized into groups receiving 1 capsule per day of Pregyn^®^ or placebo for 4 weeks. The volunteers were asked to take the capsule on an empty stomach in the morning, at least 10 min before breakfast or, in case of forgetfulness, in the evening, before going to bed and at least 2 h after the last meal. Following the first 4 weeks of treatment, the volunteers were subjected to 4 weeks of wash-out identical to the run-in period. At the end of the wash-out period, the volunteers took 1 capsule a day of Pregyn^®^ or placebo for 4 weeks according to the crossover design.

*Quantification of* Gardnerella vaginalis *in vaginal swab samples*

Real-Time quantitative (qPCR) protocols were adopted for the quantification of the *Gardnerella* *vaginalis* in vaginal total DNA targeting the lactate dehydrogenase gene, with primers Gard_LdhF, 5′-GTTATTACTGCTGGTGCTCG-3′ and Gard_LdhR, 5′-GCTCGCCAGCAATATAAGCG-3′. A gradient PCR was initially performed to standardize the qPCR conditions and also validation of the primer specificity was performed using DNA of several bacterial species (such as lactobacilli and bifidobacteria) commonly found in vagina. The qPCR amplifications were carried out in a final volume of 15 μl containing 7.5 μl of EvaGreen^®^ Supermix and 0.5 μM of each primer. Total 50 ng of vaginal DNA was used in each reaction. Samples were amplified with the following program: initial hold at 95°C for 3 min, and 39 cycles at 95°C for 30 s, 58°C for 30 s and 72°C for 30 s. Melting curves were analyzed to confirm the specificity of the amplification products. To generate the standard calibration curve for *Gardnerella vaginalis* quantification, 750 μl vaginal samples was added with different number of *Gardnerella* cells (starting from 10^6^ downwards up to 1 cell and also negative control without addition of any cells), total DNA was extracted from each preparation as described in main materials and methods. For qPCR, 50 ng DNA from each preparation (samples added with different number of cells) were used and standard calibration curve was prepared. The equation of the derived standard curve was used to calculate the correlation between Ct values and the concentration of bacterial cells per swab.

**Supplementary Table S1**. Adverse events (AE) recorded during the trial. *More than one adverse event per subject occurred.

|  | **Pregyn** | **Placebo** |
| --- | --- | --- |
| Total number of AE (N) | 39 | 55 |
| Number of AE per subject  *[Mean (standard deviation)]* | 2.17 (1.29) | 3.24 (1.82) |
| Number of AE with suspected relatedness to the treatment*  *[N (%)]* | 24 (61.5%) | 32 (58.2%) |
| Number of AE by severity level *(N)* |  |  |
| - Mild | 27 | 33 |
| - Moderate | 10 | 21 |
| - Severe | 2 | 1 |

**Supplementary figure S1**. Composition of the vaginal microbiota at baseline (V1) of the 37 reproductive-age women enrolled in the VAG-LPC 14 study. A, heatmap of log_10_-transformed relative abundances of microbial taxa found in the vaginal bacterial communities; α-diversity indices are shown as black-yellow heatmap where color indicates the value from minimum (black) to maximum (bright yellow). B, Tukey's boxplots of the bacterial taxa characterizing the identified vaginal community state types.

**A**.


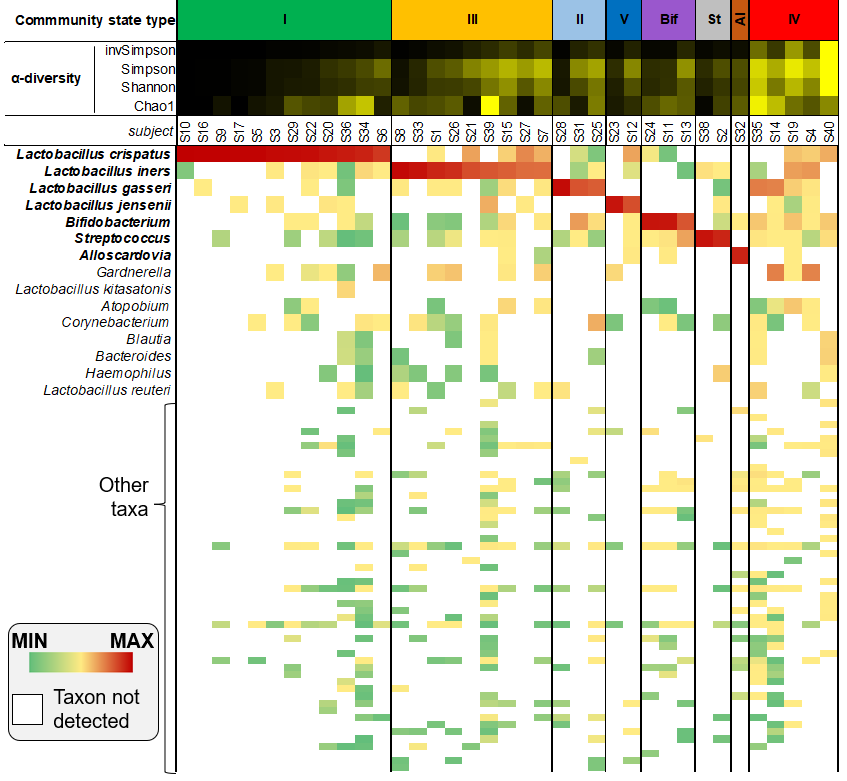


**B.**


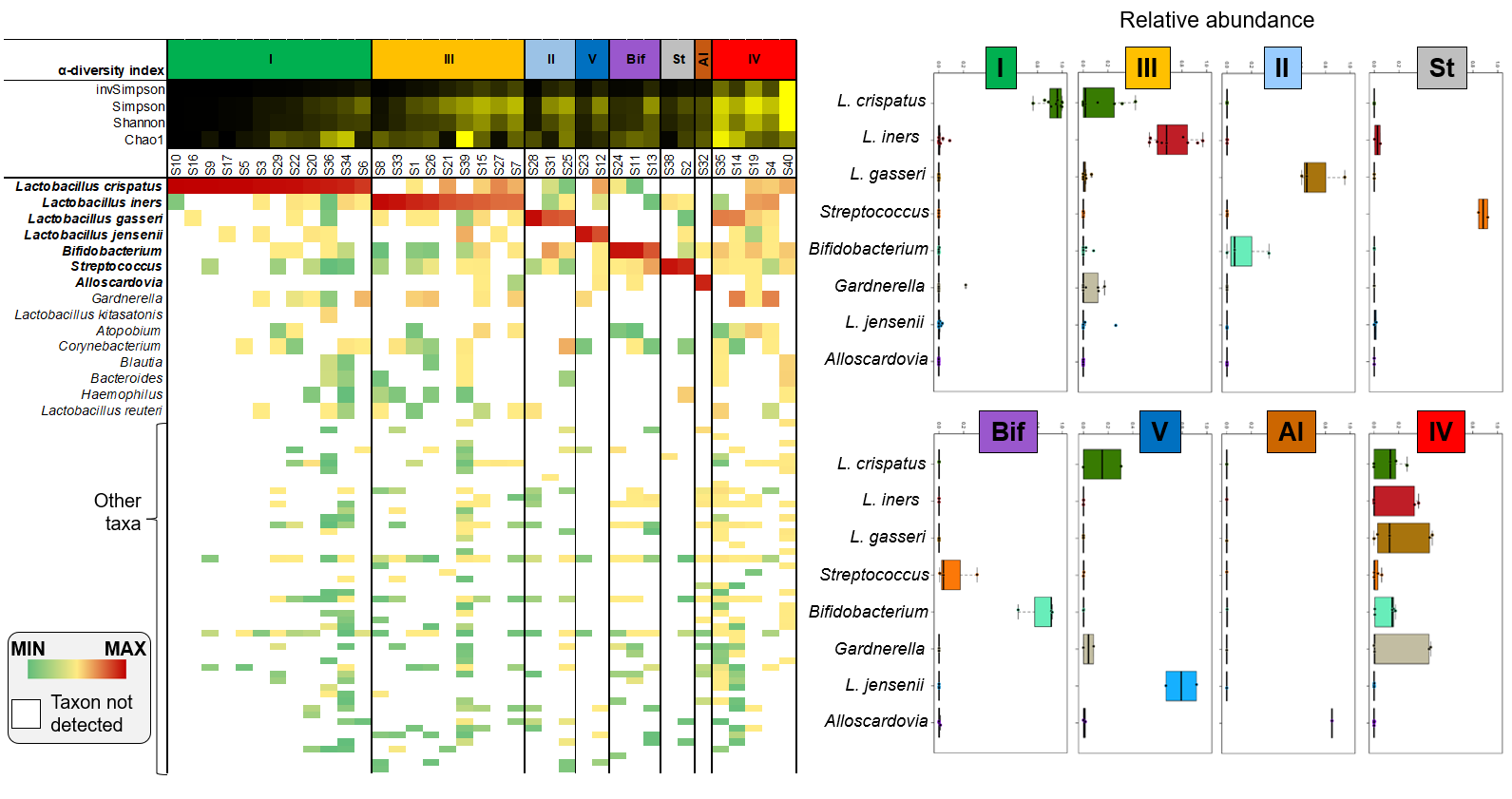


**Supplementary figure S2**. Absolute quantification by qPCR of *Gardnerella* cells in vaginal swabs. Only volunteers in whose vaginal mucosa LPC-S01 cells have been detected are shown. Lines collect samples belonging to the same volunteer. Red lines indicate increases of the cell abundance of more than 10% during an intervention; green lines indicate reductions of the cell abundance of more than 10% during an intervention. Grey lines indicate modification of the cell abundance of less than 10% during an intervention. Dotted lines indicate volunteers that were randomize to have the placebo intake phase before the probiotic intervention. Light blue lines indicate the mean. Statistics is according to repeated measure ANOVA.

**Supplementary figure S3**. Bacterial genes derived from KEGG Ortholog count prediction that were found to be significantly changed after the probiotic or the placebo phases. P values are according to the Wilcoxon signed-rank test. the black-yellow heatmap indicates median values; white boxes indicate a median of zero. Δ, deviation between the median abundances of the gene after and before the treatment; ˅, positive deviation (i.e., the gene relative abundance increased after the treatment); ˄, negative deviation (i.e., the gene relative abundance decreased after the treatment).

**Supplementary figure S4**. Results of strain-specific qPCR quantification of *L. paracasei* LPC-S01 cells in fecal samples. V1-4, time point of fecal sample collection, according to the study design reported in Fig. 1. Data are reported as log_10_ cells/g of feces. n.d., not detected; N.A., samples not available. LPC-S01 cell quantifications are reported in a yellow-red heatmap from the from minimum (yellow) to maximum (red) concentration.
